# Effectiveness of Iso-Inertial Resistance Training on Muscle Power in Middle-Older Adults: Randomized Controlled Trial

**DOI:** 10.2196/66414

**Published:** 2025-08-21

**Authors:** Aïda Cadellans Arroniz, Daniel Romero Rodríguez, Víctor Zárate, Flora Dantony, Marc Madruga Parera, Silvia Ortega Cebrian, David Blanco

**Affiliations:** 1 Department of Physiotherapy, Universitat Internacional de Catalunya, Carrer de Josep Trueta, Sant Cugat del Vallès, Barcelona, 08195, Spain, 34 935042000

**Keywords:** resistance training, power, middle-older adults, iso-inertial training, flywheel

## Abstract

**Background:**

Resistance training is commonly used to prevent the decline in muscle power associated with aging.

**Objective:**

This study aims to evaluate the effectiveness of iso-inertial (IN) training on power, physical performance, and variables associated with the risk of falls, compared to gravitational (GR) training, in physically active middle-older adults.

**Methods:**

A parallel-group, randomized controlled trial was conducted at Espai Esport Wellness Center (Granollers, Spain). In total, 44 physically active adults (age >57) were randomized 1:1 to either the IN (n=21) or GR (n=23) training groups (using R software; R Core Team). Participants completed a 6-week training program (2 sessions/week) consisting of 3 exercises (forward lunge, side lunge, and forward lunge with row). The primary outcome includes power in the eccentric phase of each exercise, evaluated using both IN and GR devices. Secondary outcomes include concentric power, physical performance, and variables associated with the risk of falls. Only outcome evaluators were blinded. We used multivariate linear regression models for the analysis.

**Results:**

In total, 27 participants completed the program (IN: n=15 IN; GR: n=12). IN training resulted in better eccentric power gains compared to GR training when assessed using the IN system, although the difference was only statistically significant for the side lunge. For forward lunge, between-group difference was 4.50 W (95% CI −2.94 to 11.94 W, *P*=.23); for side lunge, between-group difference was 9.24 W (95% CI 2.99-15.49 W; *P*<.01); and for forward lunge with row, between-group difference was 15.25 W (95% CI −0.63 to 31.13 W; *P*=.06). We observed no significant differences for the eccentric power using the GR system evaluation, concentric power, physical performance, and variables associated with the risk of falls. Both groups showed significant improvements from baseline across all outcomes.

**Conclusions:**

Although IN training appeared to result in greater power gains during the eccentric phase when assessed with the IN system, statistically significant differences were observed only for the side lunge exercise. Both training systems seemed equally effective in improving eccentric power as evaluated with the GR system, concentric power, physical performance, and reducing variables associated with the risk of falls.

## Introduction

Different indicators show that the European Union population is increasingly aging. The median age has risen from 38 years in 2001 to 41 years in 2010 and 44 years in 2020. Additionally, the proportion of older adults in the population has grown, with 21% of the population being 65 years or older in 2020, compared to 16% in 2001, representing an increase of 5 percentage points [1]. In Spain, it is projected that the percentage of the population aged 65 and over will peak at 30.5% around 2055 [2]. This, combined with the population’s progressive increase in life expectancy, has led to a growing interest in aging-related issues in recent years.

The World Health Organization recommends physical activity (PA) for the prevention of mortality, cardiovascular diseases, hypertension, specific cancers, type 2 diabetes, as well as for mental health (reducing symptoms of anxiety and depression), cognitive health, sleep quality, and potential improvements in measures of adiposity [3]. PA is also advised for managing health outcomes specific to older adults, such as fall prevention, fall-related injuries, physical function, frailty, and osteoporosis, which are consequences of the physiological decline in physical capacity [3]. Recent evidence shows that PA can reduce the fall rate in older adults by up to 23%, significantly decreasing the risk of fall-related injuries, including serious falls resulting in bone fractures, head trauma, open wounds, soft tissue injuries, or other injuries requiring medical attention or hospitalization [4].

After reaching its peak at the beginning of adulthood, muscle and bone mass begin to decrease over the years, accompanied by losses in muscle strength and power [5-7]. Recent research has revealed a significant decrease in muscle strength of 1.5%‐5% each year from age 50 [8]. For this reason, the early implementation of preventive strategies, such as promoting PA, should focus not only on older adults but also on middle-aged individuals. In addition, older adults seem to preserve the ability to produce eccentric force better than concentric or isometric force [9]. The mechanisms underlying this phenomenon appear to be both mechanical and cellular [10]. The age-related accumulation of noncontractile material in the muscle-tendon unit increases passive stiffness, which may offer a mechanical advantage during eccentric contractions [11]. This makes eccentric strength training a powerful tool for exercising or restoring muscle strength in individuals who may have a limited capacity to train at high intensities, such as older adults [9]. Additionally, some authors have suggested that eccentric contractions are more effective because they provide intense muscular work with lower metabolic expense [9]. It has also been observed that, compared to concentric-based exercises, eccentric-based exercises lead to greater maximal strength with less muscle activation and a more significant increase in muscle mass [12]. To maintain an upright position or during certain activities of daily living, such as getting up and down from a chair or walking down stairs, the lower limb muscles function in a controlled manner against the force of gravity, which requires eccentric muscle contractions [13]. Indeed, it has been suggested that maximal knee and ankle eccentric strength may be critical for safe stair descent in older adults [14].

The study of muscle power in older adults has gained interest in recent years. Power, defined as the ability to exert force in a short time interval, appears to be more closely associated with certain mobility tasks than muscle strength [15-17]. It is considered a predictor of functional capacity, as it is also linked to the execution of activities of daily living such as climbing stairs, standing up from a chair, or walking [18,19]. Previous studies have reported that power diminishes more than strength over time [12].

Resistance training is one of the main strategies used to prevent decreased functional capacity [12]. The gravity-dependent resistance exercise method, in which resistance is opposed through gravity-dependent free weights or weight machines, has the limitation that the workload applied in the concentric phase of the lengthening-shortening cycle conditions the muscle capacity progression in the eccentric phase [20]. This limitation reduces the potential of this method for generating improvements associated with eccentric training [13]. In contrast, the iso-inertial (IN) training method generates resistance through an IN device, where the inertia of a rotating mass provides the workload. Unlike the gravitational (GR) system, the IN method can generate greater force during the eccentric phase of an exercise, a phenomenon known as eccentric overload. Due to the force-generating system of IN devices, high workloads can be applied in both phases of a given action, whereas eccentric overload in GR systems can only be achieved with external assistance [13,21]. Another benefit of the IN method is that, unlike GR training, it ensures accommodated resistance and optimal muscle loading [22].

Maroto-Izquierdo et al [20] conducted a systematic review of randomized controlled trials to evaluate the effects of IN training among athletes and healthy subjects, reporting increased muscle strength, power, and muscle size compared to GR training. Nevertheless, they highlighted some methodological limitations in the studies included, such as the difficulty in isolating the eccentric phase from the concentric phase and the fact that trial participants, therapists, and outcome assessors were typically not blinded—that is, they were aware of the intervention assignment. These factors may have introduced bias into the results. More recent studies have also focused on older adults, showing improvements in postural control, maximal isometric strength, isokinetic power, and other factors in participants who trained using IN systems, compared to those who trained with GR systems [23-25].

However, the interventions in these studies focused on analytical exercises (eg, leg curl and leg extension), which did not involve large muscle groups working synergistically and in a coordinated manner. Functional exercises focus on multijoint, complex, and dynamic movements, offering a promising approach for this population [12]. These exercises are designed to improve overall functionality and facilitate the performance of daily tasks, such as lifting objects, bending down, or walking. To date, no study has compared the impact of IN and GR training on power during functional exercises or on physical performance variables in middle-older adults.

The primary objective of this study was to evaluate the effectiveness of an IN resistance training program on eccentric power compared to the same program executed with GR resistance in physically active middle-older adults. The secondary objective was to compare these 2 programs in terms of concentric power, physical performance, and variables associated with the risk of falls.

## Methods

### Trial Design and Setting

This was a parallel-group, randomized controlled trial (allocation ratio 1:1) with a blinded outcome assessment, conducted at the Espai Esport Wellness Center gymnasium (Granollers, Spain). This study was approved by the Drug Research Ethics Committee of the Universitat Internacional de Catalunya (Code: FIS-2023‐03)

We followed the CONSORT (Consolidated Standards of Reporting Trials) guidelines to produce this report [26] (the CONSORT-EHEALTH [Consolidated Standards of Reporting Trials of Electronic and Mobile Health Interventions and Online Telehealth] checklist is provided in Checklist 1).

### Participants

We recruited physically active middle-older adults (aged 57 y or older) who engaged in at least 2 days of moderate-intensity aerobic PA or strength training per week [3]. In accordance with United Nations standards, we define middle adults as those aged between 40 and 60 years, and older adults as those aged 60 and older [27]. Participants younger than 60 years of age were considered for inclusion because muscle and bone mass loss, as well as reduced physical capacity, have been reported starting at around 50 years of age [8]. The initial phase of recruitment targeted individuals aged 60 and older. However, as the recruitment period neared its end, we lowered the age threshold to maximize participation before the deadline.

Participants with osteoarticular or acute musculoskeletal injuries, or those with systemic neurodegenerative diseases, were excluded. Recruitment was carried out in collaboration with the administrative staff of the gymnasium, who contacted all members over 57 years old and invited them to participate. Participants had to sign an informed consent form before participating in the study.

### Interventions

Participants completed a 6-week program (2 sessions per week separated by at least 48 h) using either an IN or a GR resistance device. The 6-week duration was chosen based on previous studies evaluating the effectiveness of IN resistance training in older adults [19,28-30].

Each session started with a warm-up consisting of (1) 4 minutes of moderate aerobic exercise (treadmill, elliptical bike, or stationary bike), (2) active stretching exercises for 6 seconds each (hip adductors, hamstrings, gastrocnemius, quadriceps, and gluteus), and (3) 6‐8 repetitions of each of the 3 intervention exercises without resistance to familiarize participants with the exercises.

**Figure 1.**
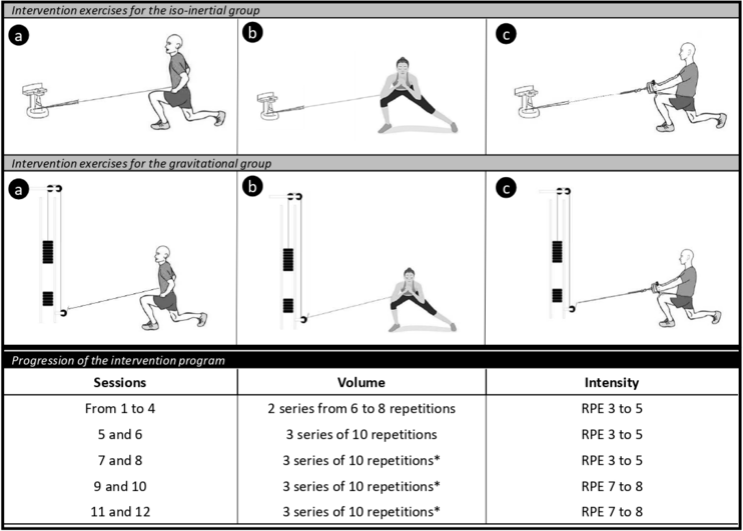
Intervention exercises and progression of the intervention program. (**A**) Forward lunge, (**B**) side lunge, and (**C**) forward lunge with row. RPE: rate of perceived exertion; *lunges performed at different distances.

Regardless of the assigned group, participants were required to perform the same 3 intervention exercises: forward lunge, side lunge, and forward lunge with row (Figure 1). Each exercise was performed individually. The IN group used the Nessinertial conical pulley (6 inertial loads), while the GR group used the MFT CSX-5000 device (weight: 3.75 kg). Each exercise was performed with both limbs. The starting limb for each exercise was randomized for each subject. Study investigators instructed participants to perform the exercises at a specific intensity based on the Borg Rating of Perceived Exertion (RPE) scale (0‐10, where 0 means no exertion at all and 10 means maximal exertion) [31]. In the first 2 weeks, participants were instructed to perform 2 sets of 6-8 repetitions at a 3‐5 RPE. In week 3, the program was increased to 3 sets of 10 repetitions at 3‐5 RPE. In week 4, volume and intensity remained the same as week 3, but participants performed lunges at 3 different distances marked on the floor (short, normal, and long), alternating between them. Weeks 5 and 6 followed a similar structure to weeks 3 and 4, but participants performed the exercises at 7‐8 RPE. A rest period of 30 seconds to 1 minute between sets was indicated. All this information is summarized in Figure 1. The load was identical for all participants, but each adjusted the execution speed of each exercise to achieve the desired RPE. More detailed information on the exercises and devices is provided in Multimedia Appendix 1. Participants were instructed not to perform any other lower limb resistance training during the intervention period, but they were free to engage in their usual aerobic exercise and upper limb resistance training. We did not monitor the training performed outside the study.

All participants were supervised by one of the study investigators or by the center’s instructors until they were shown to be autonomous. From that point onward, the sessions were performed without supervision. Additionally, participants were provided with documentary information, including images and videos of the exercises. We monitored the completion of the training program by asking participants to contact us after each completed session via WhatsApp or phone call. This communication also allowed participants to report any potential adverse events. In response, we acknowledged their feedback by congratulating them on completing the session and motivating them by providing a countdown of the remaining sessions in the training program. If participants missed a session, they were instructed to perform an extra session during the following week, ensuring a 48-hour break between sessions. Adherence was assessed by dividing the number of completed sessions by the total number of sessions expected.

### Outcomes and Instruments

Outcomes were assessed at the gymnasium facilities between 2 and 5 days before the intervention began (T0) and 2 to 5 days after its completion (T1). All the evaluation instruments described below are valid and reliable.

The primary outcome was the power in the eccentric phase (ie, eccentric power) of each exercise. This was measured using both IN and GR resistance devices, with a rotatory and a linear encoder (Chronojump Boscosystem; *r*=0.98, 90% CI 0.98‐0.99) [32], respectively. We used the free software Chronojump, in conjunction with the open hardware Chronopic V.3, to record the data. For each exercise, Chronojump provided the average power for both the eccentric and concentric phases of each repetition, calculated as the average speed multiplied by the average force for each phase. The average speed was determined by dividing the distance covered by the time taken to perform each phase. For the statistical analysis, we calculated the eccentric power for each exercise by taking the mean of the average power during the eccentric phase of the 3 best consecutive repetitions. These 3 repetitions were selected based on having the highest combined eccentric-concentric average power. Importantly, the decision to compute the average power instead of the maximum power for each phase and repetition, as well as the mean of the 3 repetitions, was aimed at providing a stable measure that minimized the impact of power peaks and fluctuations within a repetition, thus preventing atypical results or computational noise from distorting the power values.

The secondary outcomes were (1) power in the concentric phase (ie, concentric power) of each exercise, measured with both the IN and GR devices, and calculated in a manner analogous to the procedure described earlier for the eccentric power; (2) physical performance*,* assessed using the Short Physical Performance Battery (SPPB; intraclass correlation coefficient=0.839‐0.940) [33], which includes a balance test, a gait speed test, and a five-times sit-to-stand test; and (3) risk of falls, evaluated through the timed up and go test (r: 0.51‐0.78) [34]. Participants made a single attempt for each of these tests, which are further explained in Multimedia Appendix 2. For the muscle power variables, participants were instructed to perform the exercises at their maximum possible speed, while for the functional tests, we followed the verbal instructions provided in the test protocols [33,34].

We also collected anthropometric and sociodemographic data, including date of birth, weight (kg), height (cm), sex (male or female), and work status (working or retired).

### Sample Size

Given that no studies have compared eccentric power between IN and GR resistance training protocols in middle-older adults, we used a convenience sample of 44 participants, which was the maximum sample size we could achieve within the established recruitment period (November 2023 to February 2024).

### Randomization (Sequence Generation, Allocation Concealment Mechanism, and Implementation) and Blinding

The team’s statistician (DB) created the randomization sequence using R Statistical Software (R Core Team, version 4.5.0) in blocks of 4, stratified by sex and age (57‐63, 64‐70, and 71 years or older). This sequence was matched by the administrative staff of the center to the participants’ membership numbers, according to their recruitment order. As a result, both participants and researchers were unaware of the allocation until the training program started. Neither participants nor investigators monitoring the training protocols could be blinded to the allocation. However, the outcome assessors were blinded.

### Statistical Methods

For the statistical analysis, we used R Statistical Software. Initially, we presented descriptive data for all included subjects, those who completed the study, and those who were lost to follow-up. We then applied linear regression models, where the dependent variables were the change scores of each outcome (T1 minus T0), and the independent variables were the allocation group (IN or GR), baseline score, limb, and selected demographic variables (age and sex). We checked the model application assumptions (linearity, homoscedasticity, normality, and independence). Since the characteristics of the participants lost to follow-up were similar to those who completed the study, we performed a complete case analysis. We reported the adjusted between-group difference of means, along with its 95% CI and the *P* value of the comparison. We used an alpha level of .05 for statistical significance. Additionally, we calculated the intragroup improvement (both absolute value and percentage) for each study outcome. All R scripts are publicly available [23].

### Ethical Considerations

This study was approved by the Drug Research Ethics Committee of the Universitat Internacional de Catalunya (approval number: FIS-2023‐03). The protocol can be found in Multimedia Appendix 3. Participants provided informed consent to participate in the study before taking part (Multimedia Appendix 4). No identifiable features of research participants or users have been shared in any images of the manuscript or supplementary materials. No compensation was provided to subjects for participation.

## Results

**Figure 2.**
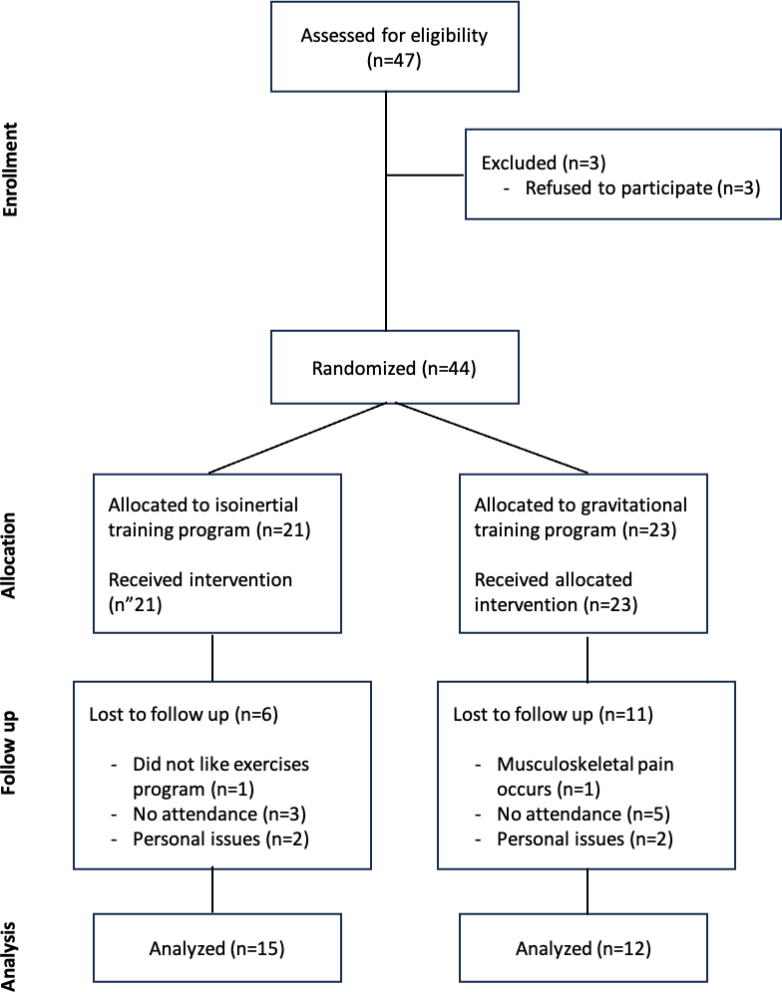
Explanation of the short physical performance battery and timed up and go tests.

Between November 10, 2023, and February 28, 2024, we recruited 47 participants. Of them, 3 refused to participate, leaving 44 participants to be randomized: 21 to the IN group and 23 to the GR group. In total, 17 patients were lost to follow-up (6 in the IN group, 11 in the GR group). The reasons for loss to follow-up were as follows: (1) failure to attend training sessions (n=8), (2) personal problems (n=4), (3) musculoskeletal problems (n=4), and (4) dissatisfaction with the training program (n=1). In the end, 27 participants completed the program (15 in the IN group and 12 in the GR group) (Figure 2). All of the completers had 100% adherence to the program.

The mean (SD) age of participants who completed the program was 63.52 (6.76) years. Of these, 16 out of 27 participants (59.2%) were female, and 11 (40.7%) were male. The baseline characteristics of all included participants were similar to those of the participants who completed the program and to those lost to follow-up (Table 1).

**Table 1.** Baseline characteristics of the participants.

	Total participants included (n=44)		Participants with full program completed (n=27)		Participants lost to follow-up (n=17)	
	IN^a^ group (n=21)	GR^b^ group (n=23)	IN group (n=15)	GR group (n=12)	IN group (n=6)	GR group (n=11)
Age (years), mean, SD	65.00 (6.36)	62.74 (6.87)	64.53 (6.90)	62.25 (6.61)	67.80 (3.56)	62.83 (7.25)
Gender						
Female, n (%)	13 (61.90)	12 (52.17)	8 (53.33)	8 (66.67)	5 (83.33)	4 (36.36)
Male, n (%)	8 (38.10)	11 (47.83)	7 (46.67)	4 (33.33)	1 (16.66)	7 (63.63)
Weight (kg), mean (SD)	68.18 (10.28)	73.09 (11.18)	68.41 (9.35)	70.67 (0.59)	1.68 (0.06)	73.90 (16.51)
Height (m), mean (SD)	1.65 (0.08)	1.69 (0.08)	1.65 (0.06)	1.67 (0.10)	1.66 (0.15)	1.66 (0.15)
Status						
Active, n (%)	10 (47.62)	15 (65.22)	8 (53.33)	8 (66.67)	3 (50)	6 (54.54)
Retired, n (%)	11 (52.38)	8 (34.78)	7 (46.67)	4 (33.33)	3 (50)	5 (45.45)

a IN: iso-inertial.

b GR: gravitational.

### Primary Outcome

Although improvements in eccentric phase power (evaluated using the IN system) were greater in the IN group across all exercises—ranging from 78.61% to 101.86%—compared to the GR group (55.43% to 86.10%), only the side lunge showed a statistically significant between-group difference (*P*<.05), as reported in Table 2. For the forward lunge, the mean eccentric power at T1 was 42.67 W in the IN group and 35.59 W in the GR group (between-group difference: 4.50 W, 95% CI −2.94 to 11.94 W; *P*=.23). For the side lunge, the mean eccentric power was 51.06 W in the IN group and 40.22 W in the GR group (between-group difference: 9.24 W, 95% CI 2.99 to 15.49 W; *P*<.01). For the forward lunge with row, the mean eccentric power was 128.39 W in the IN group and 102.41 in the GR group (between-group difference: 15.25 W, 95% CI −0.63 -to 31.13 W; *P*=.06).

**Table 2.** Intra- and intergroup analysis of power changes in the eccentric phase (in W).

Exercise	GR^a^ group				IN^b^ group				Differences between groups (95% CI)	*P* value
	T0, mean (SD)	T1, mean (SD)	Change T1-T0	% Change T1-T0	T0, mean (SD)	T1, mean (SD)	Change T1-T0	% Change T1-T0		
IN										
Forward lunge	19.12 (9.37)	35.59 (21.11)	16.47	86.10	21.14 (9.52)	42.67 (15.78)	21.53	101.86	4.50 (−2.94 to 11.94)^c^	.23
Side lunge	25.52 (12.21)	40.22 (15.25)	14.70	57.59	26.75 (12.04)	51.06 (19.37)	24.31	90.88	9.24 (2.99 to 15.49)^c^	<.001
Forward lunge with row	65.89 (45.58)	102.41 (62.81)	36.52	55.43	71.89 (40.08)	128.39 (51.49)	56.51	78.61	15.25 (−0.63 to 31.13)^c^	.06
GR										
Forward lunge	21.52 (6.36)	26.08 (8.03)	4.56	21.18	21.53 (6.50)	24.99 (5.41)	3.47	16.10	−0.51 (−2.59 to 1.58)	.63
Side lunge	19.11 (4.26)	21.75 (4.22)	2.64	13.80	19.39 (4.07)	23.03 (4.40)	3.64	18.77	0.69 (−1.20 to 2.58)^c^	.47
Forward lunge with row	42.37 (16.38)	60.08 (27.24)	17.71	41.81	42.41 (18.57)	55.58 (16.79)	13.16	31.03	−2.94 (−12.76 to 6.87)	.55

a GR: gravitational.

b IN: iso-inertial.

c Favors the IN group.

When using the GR system for evaluation, the IN group (ranging from 13.80% to 41.81% improvement) and the GR group (ranging from 16.10% to 31.03% improvement) showed similar eccentric power improvements (Table 2). For the forward lunge, the mean eccentric power was 24.99 W in the IN group and 26.08 W in the GR group (between-group difference: −0.51 W, 95% CI −2.59 to 1.58 W; *P*=.63). For the side lunge*,* the mean eccentric power was 23.03 W in the IN group and 21.75 W in the GR group (between-group difference: 0.69 W, 95% CI −1.20 to 2.58; *P*=.47). For the forward lunge with row, the mean eccentric power was 55.58 W in the IN group and 60.08 W in the GR group (between-group difference: −2.94 W, 95% CI −12.76 to 6.87; *P*=.55).

### Secondary Outcomes

The power in the concentric phase improved similarly in both the IN group (with improvements ranging from 47.81% to 81.73% in the IN system and from 5.66% to 61.23% in the GR system) and the GR group (with improvements ranging from 57.81% to 67.61% in the IN system and from 10.09% to 55.97% in the GR system) (Table 3). Additionally, improvements were observed in both groups, but no significant between-group differences were found in physical performance or in variables associated with the risk of falls (Table 4).

**Table 3.** Intra- and intergroup analysis of power changes in the concentric phase (in W).

Exercise	GR^a^ group				IN^b^ group				Differences between groups (95% CI)	*P* value
	T0, mean (SD)	T1, mean (SD)	Change T1-T0	% Change T1-T0	T0, mean (SD)	T1, mean (SD)	Change T1-T0	% Change T1-T0		
IN										
Forward lunge	21.74 (9.18)	39.51 (24.24)	17.77	81.73	26.55 (10.74)	42.29 (14.45)	15.74	59.27	−3.13 (−11.09 to 4.82)	.43
Side lunge	25.49 (12.40)	41.09 (19.00)	15.61	61.23	28.65 (12.66)	48.02 (17.43)	19.37	67.61	4.27 (−3.03 to 11.56)^c^	.25
Forward lunge with row	72.19 (48.28)	106.71 (60.33)	34.52	47.81	79.91 (44.43)	126.10 (50.11)	46.19	57.81	8.26 (−6.08 to 22.59)^c^	.25
GR										
Forward lunge	19.14 (5.43)	21.74 (4.74)	2.61	13.61	19.91 (5.27)	21.92 (5.09)	2.01	10.09	−0.20 (−1.97 to 1.56)	.82
Side lunge	17.43 (3.61)	18.42 (3.46)	0.99	5.66	17.45 (3.04)	19.60 (2.81)	2.15	12.31	0.91 (−0.12 to 1.94)^c^	.08
Forward lunge with row	43.44 (20.31)	71.44 (48.66)	28.00	64.47	52.60 (27.81)	82.04 (37.79)	29.44	55.97	7.46 (−12.42 to 27.34)	.45

a GR: gravitational.

b IN: iso-inertial.

c Favors the IN group.

**Table 4.** Intergroup analysis of physical fitness and variables associated with risk of falls changes.

Physical fitness test and outcome	GR^a^ group				IN^b^ group				Differences between groups (95% CI)	*P* value
	T0, mean (SD)	T1, mean (SD)	Change T1-T0	% Change T1-T0	T0, mean (SD)	T1, mean (SD)	Change T1-T0	% Change T1-T0		
SPPB^c^										
Score (0 to 12)	10.92 (1.16)	11.67 (0.65)	0.16	4.34	10.85 (0.80)	10.92 (1.32)	−0.07	−1.92	0.77 (−0.13 to 1.66)	.09
Balance (sec)	3.83 (0.58)	4.00 (0.00)	−0.09	−2.82	4.00 (0.00)	3.92 (0.28)	−0.31	−9.98	0.09 (−0.09 to 0.27)^f^	.31
Walking speed (sec)	3.18 (0.59)	3.09 (0.46)	−1.41	−11.54	3.18 (0.62)	2.86 (0.46)	−1.35	−10.73	0.20 (−0.11 to 0.51)^f^	.20
FTSST^d^ (sec)	12.25 (2.01)	10.84 (2.18)	−0.255	−3.07	12.66 (2.08)	11.30 (2.36)	−0.66	−7.98	−0.39 (−1.55 to 0.76)	.49
Variables associated with risk of falls										
TUG^e^ (seconds)	8.29 (1.60)	8.04 (1.02)	0.75	6.87	8.33 (1.28)	7.67 (1.20)	0.07	0.70	0.30 (−0.53 to 1.13)^f^	.46

a GR: gravitational.

b IN: iso-inertial.

c SPPB: Short Physical Performance Battery.

d FTSST: five times sit-to-stand test.

e TUG: time up and go test.

f Favors the IN group.

## Discussion

### Principal Results

IN training appeared to result in greater power gains during the eccentric phase compared to GR training when power was assessed using the IN system. However, statistically significant differences were observed only for the side lunge exercise. In contrast, when evaluated with the GR system, both types of training performed similarly. Additionally, we observed no differences in the concentric power, physical performance, and variables associated with the risk of falls. This is the first study in middle-older adults to (1) compare the effectiveness of IN and GR resistance training on the power of a coordinative action, (2) separately analyze the power in the eccentric and concentric phases of each exercise, and (3) assess power using both IN and GR devices regardless of the participant’s training group.

### Limitations

This research has several limitations. First, 38% of participants were lost to follow-up, preventing us from reaching a larger sample size. However, the characteristics of those were similar to those of participants who completed the study. Also, we did not measure other physiological parameters involved in functional and structural adaptations. Future studies could include these parameters to explore the underlying mechanisms behind the effects of IN and GR training. Although participants were warned about the incompatibility of performing lower limb strength training during the study period, the lack of monitoring other types of training outside the study may have introduced bias. Finally, even though the TUG test is a widely used tool to assess fall risk in older adults, its predictive validity remains controversial in the current literature. Therefore, our results with this variable are not entirely conclusive. A long-term follow-up of falls could provide more clarity on the issue.

### Comparison With Prior Work

Despite the growing number of studies focusing on the training and assessment of muscle power in older adults [15,16,35], only 2 studies to date have directly compared IN and GR training using this parameter. However, neither of these studies assessed power during the eccentric phase of the movement. Therefore, their results can only be compared to ours with regard to concentric power. First, Floreani et al [36] observed that IN training led to an increase in absolute maximum explosive power (MEP) during a leg press compared to GR training. Specifically, the absolute MEP improved by +10.8% for the IN group versus +0.31% for the GR group. Power evaluation was performed using a GR system. The results for the IN group are similar to those we obtained for the frontal lunge (+10.09% improvement) and side lunge (+12.31% improvement), but not for the frontal lunge with row (+55.97% improvement) when evaluating concentric power using the GR system. In contrast, the results for the GR group differ from ours. While they found little to no improvements, we observed significant gains. This discrepancy may be explained by various factors, such as differences in the outcome measure (they used MEP) and the training program. Second, the study by Sañudo et al [37] reported a 63% increase in power during a concentric action for the IN group (no data were provided for the GR group). In this case, power was evaluated using an iso-inertial system. Their results are consistent with ours, as we observed an increase in concentric power ranging from 59.27% to 67.61% for the IN group when evaluated using the IN system.

Importantly, there are 2 key differences between the 2 studies mentioned earlier and ours. First, those studies proposed training programs based on a single analytical exercise (squat or leg extension). In contrast, our protocol is functional, as it involves trunk stabilization and targets the major upper and lower extremity muscle groups, combining resistance and high-velocity exercises. This approach aims to have a greater transfer to activities of daily living. Additionally, our protocol, which includes aerobic exercise and a stretching routine in the warm-up, aligns with current position statements and consensus guidelines for PA in older adults. These guidelines recommend a multimodal approach that incorporates aerobic, strength, balance, and flexibility exercises [3].

Second, none of the 2 studies differentiated between eccentric and concentric power. Studies indicate that the preservation of muscle tension and the increased stiffness of muscle fibers in aging muscles contribute to greater active stiffness, which may enhance performance during eccentric actions. In fact, a 21.6% higher functional reserve of eccentric strength compared to concentric strength has been reported, which could be particularly relevant when initiating resistance training and rehabilitation programs for individuals with low strength levels [9,11]. Furthermore, it has been suggested that the loss of muscle power in the lower extremities may be a key factor in the limitation of mobility-related activities of daily living and in the onset of falls in older adults [16,17,38]. Given the implications of eccentric actions in terms of functionality, training, and risk of falls prevention in middle-older adults, a separate analysis of eccentric power should be included when evaluating training programs for this population.

The IN group appeared to achieve greater power in the eccentric phase than the GR group when power was assessed with the IN system. These results align with the theoretical foundation of IN training, which facilitates maximal concentric and eccentric muscle actions with brief episodes of eccentric overload [22]. Previous studies have reported that the peak force generated during the eccentric phase of movement can exceed that of the preceding concentric phase by 15%‐30%, likely due to the elastic energy storage properties inherent to the IN system [29,39]. However, our results should be interpreted with caution, as the improvements were only statistically significant in one of the 3 exercises. It is also important to note that participants’ familiarity with the IN system may have introduced bias into the results. Consequently, future studies could incorporate a longer familiarization period for all participants prior to conducting the assessments.

No differences between groups were observed in eccentric power for the GR system evaluation. This may be because of the limited power generation in the eccentric phase with this system. Previous studies in athletes have reported improvements in power favoring IN training compared to GR training; however, both groups were evaluated only with the iso-inertial system [20,40,41]. The use of a single assessment tool may have influenced the results, as some participants were already familiar with the IN system, while others were not. By assessing our participants with both systems, we were able to avoid this bias. Indeed, if the differences favoring the IR training group were due to familiarization, the GR group would have achieved more power in the GR system evaluation. This may confirm that the eccentric overload generated by IN training results in genuinely higher eccentric power values.

We found that all participants improved their physical performance and increased their score on the TUG test, with no significant between-group differences. The GR group completed the test 3.08% faster, while the IN group took 7.98% less time. The TUG test is widely recommended for detecting fall risk and is commonly used in clinical practice and geriatric research [42]. However, there is ongoing controversy regarding the determination of the optimal cutoff value for identifying fall risk [43]. For this reason, some studies consider the TUG a tool for assessing functional mobility [44]. Given the debate over cutoff points, we cannot confirm that training reduces fall risk based on the TUG test. However, we can confirm that there was an improvement in the time it took participants to complete tasks involving functional mobility.

These results align with a systematic review that reported improvements in functional performance for all participants, with a slight advantage for eccentric-based compared to concentric-based exercises [13]. Another review found improvements in unipedal balance in older adults who used an IN device compared to those using a GR one [20]. Notably, these studies are focused on analytical exercises. However, our protocol proposed a more functional approach. First, we used lunges because they involve complex functions such as decelerating a limb, force absorption, and controlling movement against an external force [45]. These functions are key components of an eccentric action [46]. In addition, all exercises emphasized trunk stabilization, which is an important factor to consider when developing interventions for middle-older adults. Some studies have reported that core stability training for older adults can improve balance and coordination and reduce the risk of falls [47].

Few studies have compared the effects of performing the same functional exercises with different types of resistance. Madruga-Parera et al [48] evaluated the effectiveness of functional exercises in handball that were biomechanically identical for the 2 study groups, with only the type of resistance changed. Our protocol was based on this approach. We suggest that future studies incorporate this methodology when evaluating the differences between training methods to minimize performance biases.

The scales used for evaluating physical performance and the variables associated with the risk of falls lacked sensitivity for the type of participants included, as we encountered a ceiling effect. For example, the participants’ mean baseline SPPB score was 10.88 out of 12 points, indicating excellent physical condition. A similar ceiling effect was noted in another study, which stated that the Berg scale could not predict the risk of falls in individuals with high levels of balance ability [49]. Therefore, we suggest that scales originally designed for middle-older adults be modified for physically active middle-older adults.

Despite the limitations mentioned earlier, there are several strengths to highlight in this study: (1) the randomized controlled trial design, (2) the pragmatic design, which is ideal for testing the effectiveness of interventions under real-life conditions, (3) the outcome evaluations using 2 different systems to minimize familiarization effects, (4) the functional training program, and (5) the inclusion of identical exercises for both groups.

### Conclusions

Although IN training appeared to result in greater power gains during the eccentric phase compared to GR training when assessed using the IN system, statistically significant differences were observed only for the side lunge exercise. There were no differences between the 2 methods in terms of eccentric power assessed using the GR system evaluation, nor for concentric power, physical performance, or variables associated with the risk of falls. Regardless of the training system, the resistance training program led to significant improvements in all outcomes.

This study helps further understand the effects of IN and GR resistance training, which can be useful for clinicians to prescribe more effective training programs for middle-older adults. Using IN devices for resistance training in middle-older adults may be a promising way to improve power during the eccentric phase of an action. Additionally, we provide strong empirical data that support orienting resistance training toward exercises involving complex coordinative actions (rather than analytical exercises) with a transfer to daily activities such as walking or going up and down stairs. This approach may increase middle-older adults’ autonomy and therefore promote healthier longevity. Finally, we warn about the ceiling effect of the current clinical evaluation tests and suggest avoiding their use in middle-older adults. Instead, we propose to use power assessments in clinical practice to monitor the improvements associated with resistance training.

## Supplementary material

10.2196/66414 Multimedia Appendix 1 Information about the execution of the exercises.

10.2196/66414 Multimedia Appendix 2 Explanation of the Short Physical Performance Battery and Timed Up and Go tests.

10.2196/66414 Multimedia Appendix 3 Study protocol approved by the ethics committee.

10.2196/66414 Multimedia Appendix 4 Informed consent.

10.2196/66414 Checklist 1 CONSORT-EHEALTH checklist.

## Abbreviations:

CONSORT: Consolidated Standards of Reporting Trials
CONSORT-EHEALTH: Consolidated Standards of Reporting Trials of Electronic and Mobile Health Interventions and Online Telehealth
GR: gravitational
IN: iso-inertial
MEP: maximum explosive power
PA: physical activity
RPE: Borg Rating of Perceived Exertion
SPPB: Short Physical Performance Battery
