# Supplementary material for: Effectiveness of Iso-Inertial Resistance Training on Muscle Power in Middle-Older Adults: Randomized Controlled Trial
Source: JMIR Aging. 2025 Aug 21;8:e66414. doi: 10.2196/66414 (PMC12370268; doi:10.2196/66414)
Supplement: Multimedia Appendix 1 [file aging-v8-e66414-s001.docx]

**Information about the execution of the exercises and the devices used.**

For the forward lunge and the forward lunge with row, the participant was placed frontally on the training device, whereas for the side lunge the participant was placed sideways on the device (homolaterally on the scrolling limb). For the forward and side lunge, we used a weight belt placed on the waist. For the forward lunge with row, we used a hand grip and the pull was performed with the upper limb homolateral to the displaced lower limb. After several repetitions to initiate the flywheel impulse, the exercises were performed to accelerate the rotation of the flywheel in the concentric action and decelerate it in the eccentric action.

For the isoinertial training system, the rope regulator was placed at the lowest possible position so that the rope rolled in a large diameter, providing more speed but less drag. For the gravitational training system, the pulley was fixed at position number 7 corresponding to a height of 75 cm above the floor. The participant was placed at a distance from the device that was determined individually as the distance where the traction string reached the maximum tension at the start of the execution of each exercise.
