# Supplementary material for: Effectiveness of Iso-Inertial Resistance Training on Muscle Power in Middle-Older Adults: Randomized Controlled Trial
Source: JMIR Aging. 2025 Aug 21;8:e66414. doi: 10.2196/66414 (PMC12370268; doi:10.2196/66414)
Supplement: Multimedia Appendix 4 [file aging-v8-e66414-s004.pdf]

7a. CONSENTIMIENTO INFORMADO

Número del estudio: FIS-2023-03
Versión del protocolo: 1
Fecha de la versión: 09/03/23
Fecha de presentación: 09/03/23
Investigador/a Principal: Dra. Aïda Cadellans Arróniz
Investigador/a Secundario/a: Dr. Daniel Romero Rodríguez, Dr. David Blanco , Dr. Marc Madruga Parera, Dra. Silvia Ortega Cebrián, Dra. Flora Dantony
Tutor/a / Monitor/a:
Departamento: Fisioterapia
Línea de investigación: "Intervencions en Fisioteràpia i Exercici Terapèutic"
Título de la investigación: Efectes del treball isoinercial sobre la potència muscular de les extremitats inferiors, l'aptitud física i el risc de caigudes en adults majors físicament actius. Assaig Clínic Aleatoritzat.

Jo, Sr./Sra.: …………………………………………………………………………..

- He rebut informació verbal sobre l'estudi i he llegit la informació escrita que s'hi adjunta, de la qual he rebut una còpia.
- He entès el que se m'ha explicat.
- He pogut comentar l'estudi i fer preguntes al professional responsable.
- Dono el meu consentiment per prendre part a l'estudi i assumeixo que la meva participació és totalment voluntària.
- Entenc que podré retirar-me en qualsevol moment sense que això afecti la meva futura assistència mèdica.

Mitjançant la signatura d'aquest formulari de consentiment informat, dono el meu consentiment perquè les meves dades personals es puguin utilitzar com s'ha descrit en aquest formulari de consentiment, que s'ajusta al que disposa la Llei Orgànica 3/2018, de 5 de desembre, de Protecció de Dades Personals i garantia dels drets digitals

Entenc que rebré una còpia d'aquest formulari de consentiment informat.

Signatura del pacient o la pacient   Data de la signatura

N.º de DNI
DECLARACIÓN DEL INVESTIGADOR O LA INVESTIGADORA

El participant que signa aquest full de consentiment ha rebut, per part del professional, informació detallada de forma oral i escrita del procés i la naturalesa d'aquest estudi de recerca, i ha tingut l'oportunitat de preguntar qualsevol dubte quant a la natura , els riscos i els avantatges de la seva participació en aquest estudi.

Signatura de l'investigador o investigadora Data de la signatura

Nom: Aïda Cadellans Arróniz
